# Visual Cascaded-Progressive Convolutional Neural Network (C-PCNN) for Diagnosis of Meniscus Injury

**DOI:** 10.3390/diagnostics13122049

**Published:** 2023-06-13

**Authors:** Yingkai Ma, Yong Qin, Chen Liang, Xiang Li, Minglei Li, Ren Wang, Jinping Yu, Xiangning Xu, Songcen Lv, Hao Luo, Yuchen Jiang

**Affiliations:** 1 Second Affiliated Hospital of Harbin Medical University, Harbin Medical University, Haerbin 150001, China; 2020021325@hrbmu.edu.cn (Y.M.);; 2 Department of Control Science and Engineering, Harbin Institute of Technology, Haerbin 150001, China

**Keywords:** meniscus injury, MRI, visual, cascaded-progressive convolutional neural network, diagnosis

## Abstract

Objective: The objective of this study is to develop a novel automatic convolutional neural network (CNN) that aids in the diagnosis of meniscus injury, while enabling the visualization of lesion characteristics. This will improve the accuracy and reduce diagnosis times. Methods: We presented a cascaded-progressive convolutional neural network (C-PCNN) method for diagnosing meniscus injuries using magnetic resonance imaging (MRI). A total of 1396 images collected in the hospital were used for training and testing. The method used for training and testing was 5-fold cross validation. Using intraoperative arthroscopic diagnosis and MRI diagnosis as criteria, the C-PCNN was evaluated based on accuracy, sensitivity, specificity, receiver operating characteristic (ROC), and evaluation performance. At the same time, the diagnostic accuracy of doctors with the assistance of cascade- progressive convolutional neural networks was evaluated. The diagnostic accuracy of a C-PCNN assistant with an attending doctor and chief doctor was compared to evaluate the clinical significance. Results: C-PCNN showed 85.6% accuracy in diagnosing and identifying anterior horn injury, and 92% accuracy in diagnosing and identifying posterior horn injury. The average accuracy of C-PCNN was 89.8%, AUC = 0.86. The diagnosis accuracy of the attending physician with the aid of the C-PCNN was comparable to that of the chief physician. Conclusion: The C-PCNN-based MRI technique for diagnosing knee meniscus injuries has significant practical value in clinical practice. With a high rate of accuracy, clinical auxiliary physicians can increase the speed and accuracy of diagnosis and decrease the number of incorrect diagnoses.

## 1. Introduction

Meniscal injuries are a common result of everyday exercise, and the majority of meniscal surgeries are still partial meniscectomy [1,2]. The diagnostic process prior to performing these procedures, however, is difficult, particularly the interpretation of MRI images of the meniscus [3,4,5,6]. Some injuries may be visible on MRI scans but difficult to see during arthroscopy. Surgery has been shown in several studies to decrease the likelihood of future knee osteoarthritis (KOA), enhance knee mobility, and enhance patients’ quality of life and long-term satisfaction [7,8,9,10,11]. Therefore, the early and accurate diagnosis of meniscus injury, along with timely treatment, is crucial.

Knee magnetic resonance imaging (MRI) is a valuable imaging diagnostic tool for knee disease examination. MRI has been repeatedly shown to detect pathology in the meniscus and cruciate ligament. This diagnosis method has a high level of accuracy and is frequently used to determine which patients require surgery. The meniscus have a low moisture and fat content, and MRI measures low signal intensity on T1, T2 sequences in the knee joint. In the coronal view, sequential imaging of the medial and lateral meniscus reveals a hypointense, triangular structure with a sharp tip at the apex. The anterior and posterior roots of the meniscus are triangular in shape on sagittal imaging, while the more central position is bowknot-shaped [12,13,14,15].

Furthermore, the negative predictive value of knee MRI is close to 100%, so MRI can be used as a noninvasive method to eliminate meniscus injury; however, the diagnostic accuracy of MRI may be decreased in several circumstances, such as (1) observer inexperience and bias, (2) small partial or incomplete tears, (3) imaging artifacts, (4) an incomplete MRI study, and (5) the presence of concomitant injuries. However, due to the number of MRI images captured for each knee and the level of detail, knee MRIs require a lot of time, and doctors may have differences in diagnosis due to subjectivity [8,16,17]. Deep learning methods, on the other hand, can automatically learn feature images, which makes them ideal for modeling the complex relationship between medical images and their diagnosis.

Recent advances in medical imaging include classification of skin cancer [18,19], detection of diabetic retinopathy [20], and detection of lung nodules [21]. A convolutional neural network is superior to traditional image analysis methods and has been widely adopted in the intelligent recognition of medical images. In MRI, the shape of the meniscus, any associated tears, and the severity of the injury are visible. Therefore, we attempted to diagnose meniscus injury by applying a convolutional neural network to knee MRI images [12]. The expectations include aiding doctors, speedier diagnosis, and improved diagnostic precision.

In this study, we describe a completely automated convolutional neural network that is used for the identification of MRI meniscus injuries of the knee and to visualize the results, evaluate the performance of the model and assess the accuracy of C-PCNN in assisting physician diagnosis, with the aim of improving diagnostic precision and speeding up the diagnosis process for doctors.

## 2. Method

This study was approved by our hospital ethics committee. Written informed consent was obtained from all subjects for retrospective data analysis.

### 2.1. The Experiment Design

**Figure 1:**
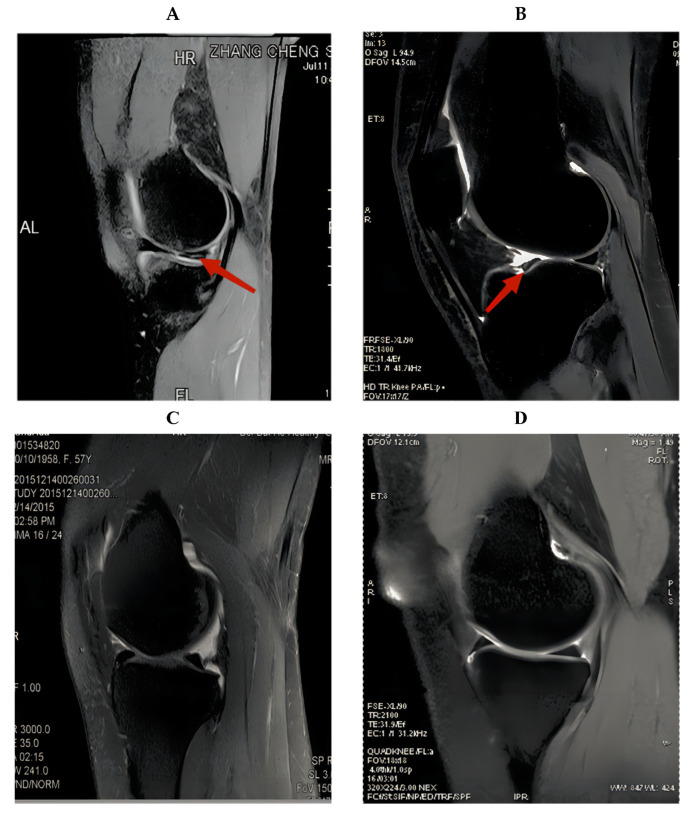
MRI images of knee joint with meniscus injury. (**A**) The posterior root injury of the meniscus. (**B**) The anterior root injury of the meniscus. (**C**,**D**) MRI images of knee joint without meniscus injury. The arrows point to the meniscus injury.

In this study, we constructed a cascade-progressive convolutional neural network (C-PCNN). The research was based on knee arthroscopy reports and MRI reports. The objective was to compute the diagnostic performance of the physician with C-PCNN diagnostic judgment and the results of C-PCNN. Because T2 MRI of the knee provides more information to detect meniscus injury, sagittal T2 knee images were employed in this investigation. The knee T2 MRI pictures contained fat suppression images [12]. Concurrently, five sports medicine professionals used C-PCNN to retrospectively evaluate the knee MRIs of clinical patients and they used C-PCNN to assess meniscus injury. We then compared the performance of physicians using convolutional neural networks (Figure 1). All meniscus-tear patients underwent arthroscopic knee surgery.

The primary outputs of C-PCNN comprise the diagnosis of meniscus injury, the lesion area, and the visualization of the lesion area. Although there are numerous classifications of the tearing direction of meniscus injury, horizontal meniscal tears are the most common. The availability of other directions of meniscal tears for training was minimal; therefore, the experiment did not define the classification of meniscus injuries. In addition, the direction of meniscus tears can be determined based on the specific visualization data.

### 2.2. Data Collection

**Figure 2:**
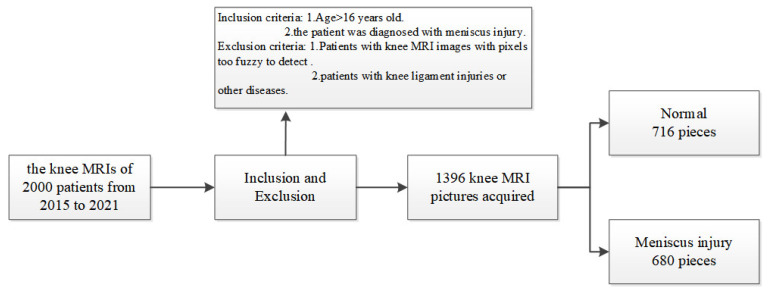
Knee MRI data collection process. A total of 2000 knee MRI images were collected. Some images were blurred and indistinct, or multiple knee injuries were excluded. A total of 716 normal images and 680 images of meniscus damage were collected.

**Figure 3:**
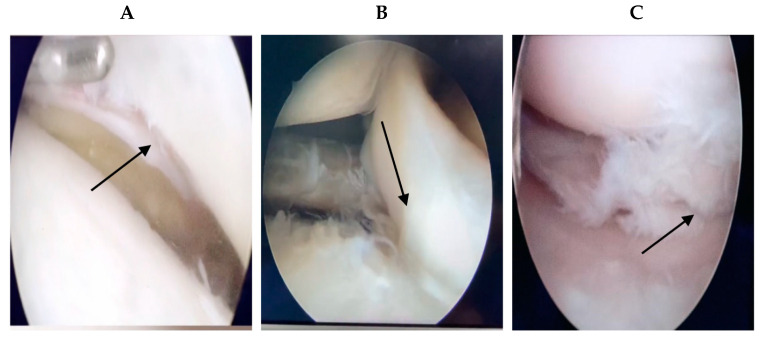
The intraoperative arthroscopic images of meniscus injury. Intraoperative diagnosis was made according to the arthroscopy of knee joint. The arrows point to the meniscus injury.

Patients who underwent surgery in our hospital and healthy patients who underwent MRI screening at our hospital were included. As there was no standard format for all MRI patient data, we selected the knee MRIs of 2000 patients from 2015 to 2021, excluding pixels that were too blurry to detect and patients with knee ligament injuries or other disorders. A total of 1396 knee MRI pictures were acquired, 716 of which were normal (Figure 2). For the remaining 680 pieces depicting meniscus injury, the age range was between 17 and 62 years (17–62 years). For all patients with meniscus injury in our hospital, the chief physician performed arthroscopic surgery, and the intraoperative diagnosis was the same as the MRI diagnosis of the radiologists. If a problem of knee joint meniscus and clean-up resection occurred, meniscus stitches were used. Figure 3 shows the intraoperative arthroscopic images of meniscus injury (Figure 3).

### 2.3. Meniscus Anatomy and Image Preprocessing

Performing MRI of the knee joint is an efficient approach for diagnosing the meniscus. The knee menisci are two crescent-shaped discs of fibro cartilage that are located between the surfaces of the femur and tibia in the medial and lateral compartments of the joint. At the cross section, the normal meniscus is wedge-shaped, with a flat surface facing the tibia and a concave surface facing the femur [4,15]. The meniscus’ fibrocartilage contains little water and fat. On a coronal picture, the anterior and posterior corners of the meniscus exhibit a low-intensity curve structure that is related to the meniscus root bone and the joint capsule perimeter. The anterior and posterior corners of the meniscus are triangular in a sagittal view. At the level closer to the middle of the knee joint, the anterior and posterior corners are shaped like a bow tie. When a meniscus injury occurs, MRI of the knee will reveal a high-signal, white and thin shadow in the meniscus region, proving that meniscus damage has occurred. To identify the tear of the meniscus, the location of the meniscus injury is first detected, followed by the extraction of the picture of the meniscus injury for feature extraction. Visual output was conducted. The knee MRI data we collected included images in different resolutions. There are background areas around the knee that do not contribute to the diagnosis. Therefore, the MRI images were clipped to be 640 × 320, 1280 × 640, and 2560 × 1280 ppi.

### 2.4. C-PCNN Architecture

**Figure 4:**
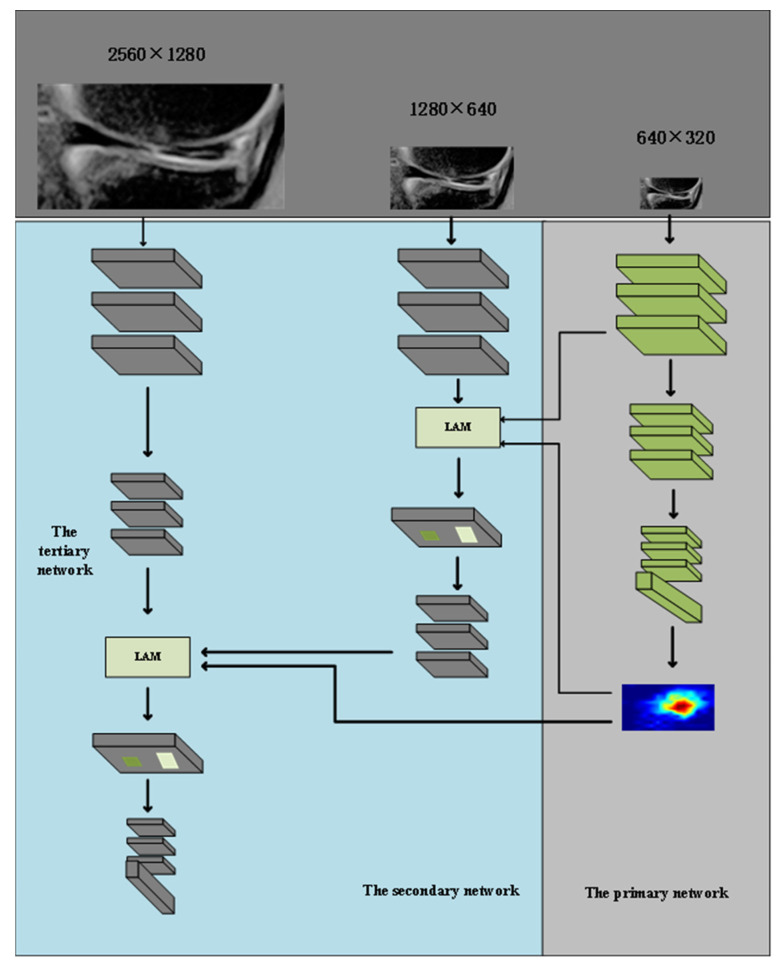
Structure of the C-PCNN. C-PCNN contains three levels including the primary network, the secondary network, and the tertiary network. Each corresponds to three resolutions (640 × 320, 1280 × 640, and 2560 × 1280). The secondary network and the tertiary network focus on the characteristics of the lesion area. The features of different resolutions and lesion localization maps are fused through LAM.

To diagnose lesions with high accuracy, we developed a cascade-progressive convolutional neural network (C-PCNN) based on the pyramid network [22,23]. The cascaded-progressive convolutional neural network consists of three components: the primary network, the secondary network, and the tertiary network, which correspond to three resolutions (640 × 320, 1280 × 640, and 2560 × 1280, respectively). Using Grad-CAM, the primary network developed the meniscus damage localization map. The lesion attention module (LAM) combined the features of the low-resolution image, the meniscus injury mapping generated by the primary network, and the high-resolution characteristics. The cascade-progressive output branches of the secondary and tertiary networks were used to obtain the final diagnosis results. The output branch of the high-resolution network is used to obtain the final diagnosis result. The output branches of the primary network were used to obtain the meniscus damage localization map and the lesion’s location under the supervision of image annotation. The final diagnosis results were obtained by cascade-progressive output branches of the secondary and the tertiary networks. In the tertiary networks, the focal point is on the lesion features of the meniscus injury site, and the categorization and diagnosis of meniscus injury is eventually achieved. Consequently, the meniscus injury localization map generated by the primary network is used to weight the features in the tertiary network, and the specially designed attention module combines the low-resolution features generated by the primary network, the high-resolution features generated by the secondary and tertiary networks, and the meniscus injury localization map (Figure 4).

C-PCNN retrieves features from images with varying resolutions. In this study, the three network layers are referred to as the primary network, the secondary network, and the tertiary network. Each of the three networks is constructed using ResNet50 modules. ResNet50 is a ResNet family network with a number of network layers and model sizes ideal for medical imaging activities. Table 1 depicts the modules used in the planned network. Specifically, the primary net is configured as {conv_1, block_1, block_2, block_3, block_4, fc}, the secondary net is configured as {conv_1, block_1, block_2}, and the tertiary network is configured as {conv_1, conv_2, block_1, block_2, block_3, block_4, fc}.

C-PCNN performs features cascading and progressive propagation from the primary network to the advanced network. Different resolutions of images are directly added into the network. To accommodate the different-sized feature maps, additional independent convolutional layers or pooling layers must be added. After block_1, the primary and secondary networks’ characteristics are combined. After block_2, the secondary network and tertiary network’s features are merged. The advanced network receives the conv_2 module. The size of the lesion feature map was halved to ensure that fusion features had the same size.

### 2.5. Meniscus Localization

To find the meniscus, a meniscus injury localization map was generated in the primary network using weakly supervised localization. We selected the improved Grad-CAM for weakly supervised localization, which was formed by the sequential inclusion of a convolutional layer, global average pooling layer, and Softmax prediction layer at the end of the network. Although it can accentuate the most distinguishing features of an object, it is not the only method for achieving this effect. However, the CAM approach modifies the structure of CNN, whereas the upgraded Grad-CAM can generate visual data from any CNN-based network structure without modifying the network architecture. The meniscus damage localization map in the principal network was generated with the Grad-CAM approach. Thus, the location of each meniscus was determined. In order to give intuitive categorization results, the suggested method can not only determine whether the meniscus is damaged or not, but also determine the location of the injury.

Firstly, the primary network inputs the 640 × 320 image *I*_320_, and the *l*-th layer feature map $f_{i}^{l}$ can be obtained by:

$$\;f_{i}^{l}]=\mathrm{Prinet}\left(I_{320}\right)$$

We focus on the lesion area, where *l* represents the third convolutional layer in block_3, and *i* is the *i*-th channel of the feature map. Then, the gradient of the feature map is calculated, and by going through the global average pooling (*GAP*) layer, the weight of feature map is obtained:

$$\alpha_{i}^{l}=GAP\left(\frac{\partial y_{\mathrm{low}}^{c}}{\partial f_{i}^{l}}\right)$$

The final step is a linear mapping of the weights and the feature graph, which is obtained using the ReLU activation function to obtain the heat map.

$$L_{Grad-CAM}=\mathrm{ReLU}\left(\sum_{i=1}\alpha_{i}^{l}f_{i}^{l}\right)$$

### 2.6. Weighting and Visualization of Features

**Figure 5:**
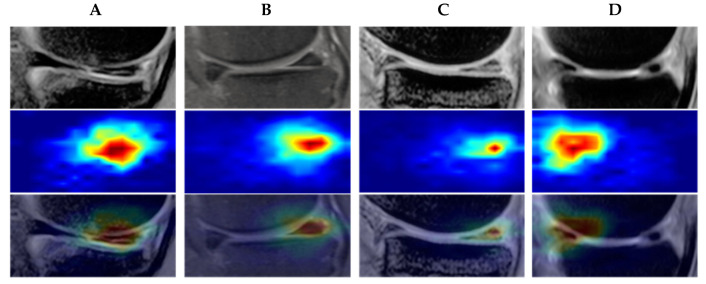
Example of lesion localization heat maps in the data set. The first row (**A**–**D**) shows the original images in the data set, the second row shows the images of heat maps of the lesion, and the third row shows the images of the feature visual output results.

This section explains how to visualize and concentrate on the characteristics of meniscus injury. The central concept of this diagnostic method is the cascade of primary, secondary, and tertiary networks based on the location of the meniscus injury. Grad-CAM obtained the lesion activation map, and the next step was to enhance the lesion feature set and lesion features. We created a LAM with two primary functions. The feature attention map between high-level and low-level network features is constructed initially. The weighting of advanced features based on meniscal injury mapping is a second function. Consequently, the LAM input consists of three components: the meniscal injury localization map, low-level network features, and high-level network features. LAM produces the weighted advanced network feature image, the meniscus injury location map generated by Grad-CAM for the low-level network, and the high-resolution network feature map. After linear transformation, new features are obtained, which are then combined with features from different levels. A 1 × 1 convolutional layer and a softmax function were used to compute the fused features for the feature weighted attention map. For the meniscus injury localization map weighting, the lesion area should be given equal weight. The meniscus injury localization map had pixels measuring 640 × 320. It was then multiplied to produce the positioning weighted attention map. Multiplying the linearly transformed feature of the feature map generated by the advanced network with the weighted attention map of the location map yields the weighted high-resolution feature. Figure 5 shows an example of a meniscus injury localization heat map in the data set. The original meniscus injury images, lesion localization images and visual outputs are shown from top to bottom. (Figure 5).

### 2.7. 5-Fold Cross Validation Method

**Figure 6:**
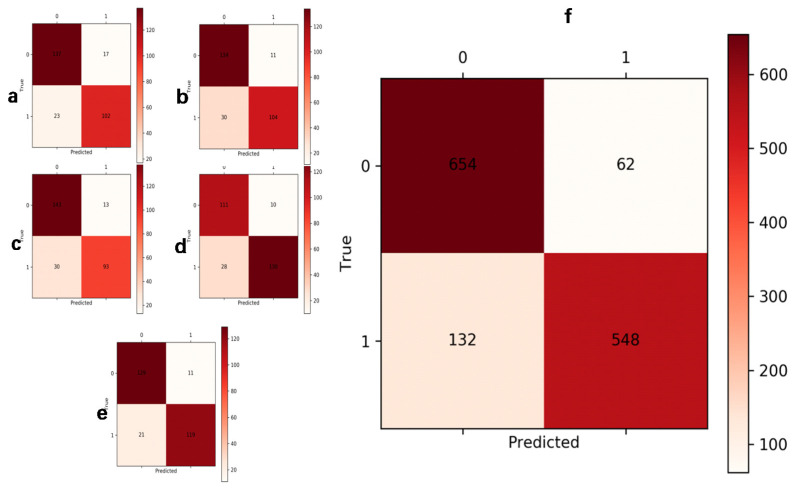
(**a**–**e**) The results of each cross-validation, and (**f**) is the total results of five-fold cross validation.

We divided the images of all 1396 patients into five parts labeled as K1, K2, K3, K4, and K5. Firstly, K2, K3, K4, K5 are used as the training set to train, and the model logist_model_Flod1 is obtained. K1 is used as the test set to test, and the accuracy logis_precise_Flod1 is obtained. The model logist_model_Flod2 is obtained by training K1, K3, K4, K5 as the training set, and the accuracy logis_precise_Flod2 is obtained by testing K2 as the test set. The model logist_model_Flod3 is obtained by training with K1, K2, K4, K5 as the training set, and the accuracy logis_precise_Flod3 is obtained by testing with K3 as the test set. In this way, the five parts are used as test sets for verification, and the average value of the five evaluation results is taken (Figure 6).

The advantage of this method is that randomly produced subsamples are frequently utilized for training and verification simultaneously, and the results are only confirmed once each time, which improves the accuracy. Cross-validation may utilize limited data effectively, and assessment results can be as close as feasible to the model’s performance on the test set, which can be used for model optimization.

### 2.8. Comparison of Different Networks

**Figure 7:**
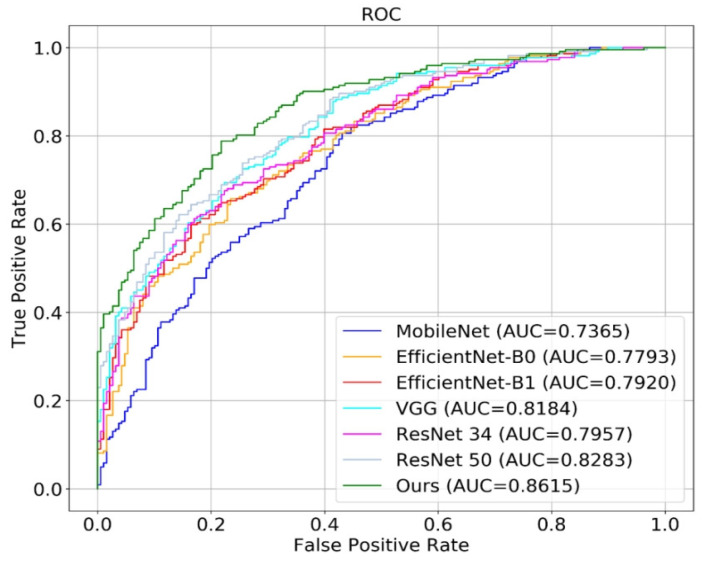
The ROC curve and AUC value for basic network and C-PCNN. Ours stands for C-PCNN. The six basic networks are EfficientnetB0, EfficientnetB1, MobileNet, ResNet34, ResNet50 and VGG.

**Figure 8:**
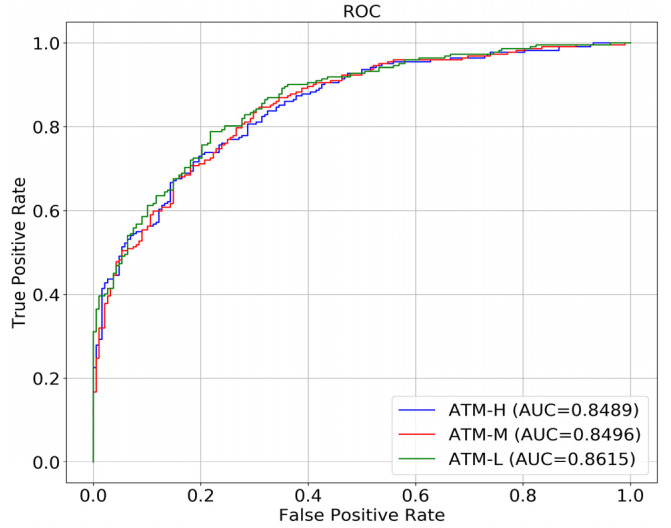
The ROC curve and AUC value for three networks. ATM-H represents the tertiary network of the high-resolution network, ATM-M represents the secondary network of the medium resolution network, and ATM-L represents the primary network of the low resolution network.

We conducted a comparison of the performance of single networks, C-PCNN, and different underlying networks. The single network selects the primary network, the secondary network, and the tertiary network in the C-PCNN. Additional convolution neural networks include EfficientnetB0, EfficientnetB1, MobileNet, ResNet34, ResNet50 and VGG, as shown in Table 2 and Figure 7 and Figure 8. ATM-L, M, and H, respectively, represent the primary network, the secondary network, and the tertiary network. By performing a comprehensive comparison of Figure 7 and Figure 8, we observed that, excluding C-PCNN, the primary network performs best. According to the results of Figure 8, the single network with high resolution has the lowest AUC. We believe that with the improvement of image resolution, network performance will gradually decline. The network can achieve satisfactory results under low resolution conditions, but its performance will decline rapidly as the image resolution gradually increases. We analyzed this outcome, suggesting that with the increased image resolution, the image resolution of the non-diseased area also increases, which leads to the increase in the interference of the non-diseased area of the network, and results in the decreased network performance. Among the different baseline networks, ResNet50 demonstrated the best performance.

### 2.9. The Diagnose of Physicians

We searched for three orthopedic attending physicians with nine, eight and eleven years of experience, and two chief orthopedic physicians, although none of them were the chief physicians of knee MRI meniscus injury or had ever viewed the 1396 images. We divided the 1396 images into seven groups, each containing 200 images, and randomly assigned the images to five individuals, one group per individual. The diagnostic results of MRI diagnosis and intraoperative diagnosis were used as reference standards to calculate the accuracy rate of five doctors in diagnosing knee meniscus injury. With the aid of the C-PCNN, three attending physicians re-diagnosed 200 films previously diagnosed by the chief physician, and graded and labeled them accurately. Finally, we found that the speed and accuracy of knee MRI image recognition by three orthopedic attending physicians improved.

### 2.10. Statistical Methods

Prism version 9.0 was used to conduct statistical analyses. General descriptive statistics were used to compare the sensitivity, specificity, and accuracy of the C-PCNN with the gold standard of intraoperative arthroscopic diagnosis and the clinician’s diagnosis. The probability of using C-PCNN to diagnose a meniscal tear was analyzed using a receiver operating characteristic (ROC) curve, and the area under the ROC curve (AUC) was calculated. Simultaneously, the accuracy of the coronal diagnosis of anterior and posterior meniscus injury horns was determined. Using C-PCNN, before and after using convolutional neural networks, a subgroup analysis was conducted to compare the performance of various physicians and to determine the clinical significance of convolutional neural networks.

## 3. Result

We used intraoperative arthroscopic diagnosis and MRI diagnosis as reference to validate the accuracy of convolutional neural networks in the diagnosis of meniscus injury, and compared the performance of a single network, a hierarchical network, and the C-PCNN (Table 2, Figure 7 and Figure 8). All experimental outcomes are displayed in the table and figure. The network performance deteriorates as image resolution increases. In the cases of low resolution, the network achieves satisfactory results, indicating that although the diseased area is more distinct in the high-resolution image, the interference of the non-diseased area is also more severe, resulting in poor results for the convolutional neural network. However, when the C-PCNN is applied, the diagnostic accuracy approaches 89.8%, AUC = 0.86, demonstrating that image feature extraction and fusion with several resolutions can significantly enhance diagnostic accuracy. The accuracy of the C-PCNN in recognizing the anterior root injury was 85.6%, while the accuracy of the diagnosis and recognition of the posterior root injury was 92%. Our subgroup analysis compared the accuracy of attending physicians and senior chief physicians using the C-PCNN, and it was found that attending physicians achieved an accuracy comparable to that of senior chief physicians with the aid of this neural network (Table 3). During the test, we analyzed the time required for diagnosing MRI images. The three attending physicians had an average diagnosis time of 80 min, while the two chief physicians had an average diagnosis time of 65 min. The test was conducted on a server equipped with two Intel E5 2678 CPUs and four NVIDIA GTX 1080Ti GPUs. The time taken to process a single image during the test was 1.5 s. However, with the assistance of C-PCNN (presumably a computer-aided diagnosis system), the diagnosis time was significantly reduced. The average diagnosis time for the three attending physicians decreased to 60 min, and for the chief physicians, it decreased to 55 min.

## 4. Discussion

In this study, we created a CNN model to identify the presence and type of meniscal tears using MRI images as input data. Our developed C-PCNN performed well in determining whether meniscal tears existed or not. Although our algorithm was trained on 1396 images, larger data sets help the algorithm to perform better. A substantial amount of annotated image data, which continually improve during training and testing, is needed for real clinical application. One approach may be to mine disease course and image reports through big data because a significant amount of data annotation and accurate diagnosis is one of the limitations of obtaining large data sets. By establishing and continually growing the database, an algorithm that is more accurate and efficient can be constructed.

Of the 1396 images, 716 displayed normal knee anatomy, while 680 depicted meniscus injury. Overall, 298 of the 680 MRI knee meniscus injury images depicted an anterior horn injury. C-PCNN had an 85.6% accuracy rate in identifying anterior horn injury. In 382 instances of posterior horn injury, the accuracy of C-PCNN diagnosis and recognition was 92%. We believe that the difference in MRI sensitivity between anterior and posterior angles meniscus tears may be the primary reason for the difference in the accuracy of the anterior and posterior angles of the sagittal meniscus. Because the angle after injury is greater on the inside, the sample selection supports this. After injury of the meniscus, anterior horn injury and a higher incidence of the lateral meniscus injury occur. In the sample selection, anterior corner injury of the lateral meniscus is more prevalent; the sensitivity and specificity of MRI for medial meniscus injury were 92% and 90%; for lateral meniscus tear, the sensitivity and specificity of MRI were 80% and 95% [15,24]. Although MRI has excellent specificity and sensitivity for medial meniscal tears, there is lower sensitivity in the detection of lateral meniscal tears [14]. Therefore, in the recognition process, both readers and the convolutional neural network may be incorrect, and the diagnosis error may be resultant of the low sensitivity of the MRI to the lateral meniscus.

The error is large when relying only on MRI of the local lesion to determine the torn meniscus injury direction, because the MRI images are continuous, and the level of meniscus injuries is judged in succession, while coronal and sagittal sequences are simultaneously utilized to determine the specific location of the meniscus injury and the classification of the meniscus injury. Due to the fact that the direction of the torn meniscus injury is multifaceted, there are nine types of classification of damage tear, and the classification of different incidences makes sample collection challenging; thus, the convolution neural network cannot currently guarantee accuracy of the nine types of meniscus injury classification. Therefore, we chose convolutional neural networks to assist doctors for accurate diagnosis. Our study found that the combination of AI and human experts is more accurate at diagnosing meniscal tears than either human experts or AI alone. After the convolutional neural network determines whether or not there is an injury, the doctor determines the precise classification. The localization map of meniscus injury can be used to improve the characteristics of lesions, the direction of meniscus injury can be determined by doctors, and the rate of doctor’s diagnosis can be added to increase the precision of diagnosis. This parallels the argument made by Kyle N. Kunze et al. [17]. In other words, it may be possible in the future to achieve rapid initial screening using convolutional neural networks and to strengthen the characteristics of lesions through the visualization of lesion sites in order for professional sports medicine physicians to classify meniscus injuries in greater detail.

We also compared our work with that of other groups that have conducted similar studies. V. Roblot et al. [25] utilized mask-RCNN to diagnose meniscus damage with AUC_Position_ = 0.92 and AUC_Orientation_ = 0.83. The meniscus was first located. The photographs were then split into four groups (background, meniscus untorn, meniscus horizontal torn, meniscus vertical torn). The AUC and accuracy drop when the direction of the meniscus tear is separated into two categories. If additional tear directions of the meniscus are categorized, the accuracy may decrease further. Hyunkwang Shin et al. [26] evaluated the meniscus injury using CNN, first localizing the injury and then classifying it as a horizontal tear (normal/horizontal), compound tear (normal/complex), radial tear (normal/radial), longitudinal tear (normal/longitudinal tear), or lateral tear (normal/longitudinal tear). The AUCs for detecting tears in the medial meniscal and lateral meniscal were 0.888 and 0.817. Regarding the ability to differentiate the type of meniscal tear, the AUCs for horizontal, complicated, radial, and longitudinal rips were 0.761, 0.850, 0.601, and 0.858. The classification performance is better than the convolutional neural network model of V. Roblot et al. [25], which we believe may be related to the accuracy of MRI images and the selection of MRI images. Benjamin Fritz et al. [27] studied a small sample size of 100 instances to compare DCNN with doctors and intraoperative observations. The sensitivity for medial meniscus injuries was considerably different between readers and the DCNN (*p* = 0.039), although all other comparisons exhibited no significant differences (*p* > 0.092). Inter-reader agreement was excellent for the medial (kappa = 0.876) and adequate for the lateral (kappa = 0.741) meniscus. It is thought that there is no substantial distinction between DCNN and its readers.

Compared to the three aforementioned studies, we have an advantage in this test as we did not impose restrictions on the MRI scan specifications, allowing for inconsistent and heterogeneous data, which we believe is more representative of clinical practice. Previous studies have indicated that the accuracy of our C-PCNN could be improved if all MRI images are obtained from the same machine or institution. In such cases, our C-PCNN achieved an accuracy of 89.8% and an AUC value of 0.86 in diagnosing meniscus injuries. The accuracy in terms of diagnosis and localization did not significantly differ from the studies conducted by Benjamin Fritz et al. and Hyunkwang Shin et al.

With the inclusion of lesion features, the heat map generated by our C-PCNN facilitated an easier determination of the direction of meniscus tears. Unlike the aforementioned studies, our C-PCNN primarily serves as an assistive tool for doctors, aiming to improve their accuracy, reduce diagnosis time, and expedite the learning curve for MRI diagnosis of meniscus injuries. Consequently, our C-PCNN is better suited for clinical use.

In contrast to radiology, the advantage of our C-PCNN lies in its utilization of T2 lipostatic images of the knee instead of requiring full-layer sequences of knee MRI. Additionally, the diagnosis time of our C-PCNN is shorter. The C-PCNN reduces the reliance on manual rules and feature engineering. This makes our method more flexible and adaptable to different types and degrees of injuries. In contrast, traditional radiological methods often rely on human expertise and professional knowledge for interpretation and diagnosis, which can be influenced by subjective factors. While MRI exhibits high sensitivity for tears, it may not be as effective in detecting fragments and is better at diagnosing medial meniscal lesions compared to lateral ones. However, by combining arthroscopic diagnosis with an improved and trained C-PCNN model, it is possible to identify more detailed information and reduce the probability of false-negative diagnoses in meniscus injuries.

The recognition of knee meniscus injuries by convolutional neural networks (CNNs) and the application of MRI images in clinical practice require the realization of multi-source and multi-layer image fusion. The diagnostic capabilities of CNNs also need to evolve from single image diagnosis to provide a comprehensive evaluation of continuous MRI outputs. This will enable artificial intelligence to simulate the diagnostic process of doctors, while observing and assessing meniscus injuries at a three-dimensional level.

Moreover, CNNs can be utilized to integrate clinical signs, symptoms, surgical methods, and complications. This integration will contribute to more accurate classifications. Additionally, the development of clinical prediction models can aid in determining the necessity of surgery and predicting the postoperative prognosis for patients. This direction of research represents our future focus, so that CNN can be better applied in the clinic, not only in disease judgment, but also in prediction, selection of surgery, prediction of postoperative complications and other directions.

## 5. Conclusions

This paper demonstrates the effectiveness of fusing a high-level network and low-level network to improve diagnosis performance. CNN is capable of learning multi-level characteristics from knee MRI images by detecting low-level network lesions and extracting complex high-level characteristics from high-level networks.

The detection of a torn meniscus can be performed automatically. The precision of our C-PCNN is comparable to that of musculoskeletal specialists. In addition, with the aid of cascades and progressive convolutional neural networks, the diagnostic rate and precision of physicians will be enhanced.

## Figures and Tables

**Table 1:** The modules in the C-PCNN, conv: a convolution layer or pooling layer; block: a residual block module; fc: the full connection layer for output the final result. The parameters in the layer are kernel size, channel number and stride.

Name	Layer
conv_1	7 × 7, 64, 2 3 × 3 maxpool, 2
conv_2	3 × 3, 64, 2
block_1	1 × 1, 64 3 × 3, 64 × 3 1 × 1, 256
block_2	1 × 1, 128 3 × 3, 128 × 4 1 × 1, 512
block_3	1 × 1, 256 3 × 3, 256 × 6 1 × 1, 1024
block_4	1 × 1, 512 3 × 3, 512 × 3 1 × 1, 2048
fc	average pool 2-d fc softmax

**Table 2:** Comparison between different convolutional neural networks. tp: true positive; tn: true negative; fp: false positive; fn: false negative.

Model	ACC	Pre	Recall	Spe	AUC	Fl-Score	tp	tn	fp	fn
ATM-THREE	0.8468	0.8624	0.8206	0.8757	0.8489	0.841	558	627	89	122
ATM-SECOND	0.8532	0.8705	0.8206	0.8841	0.8496	0.8448	122	633	83	122
C-PCNN	0.861	0.8984	0.8059	0.9134	0.8615	0.8496	548	654	62	132
EfficientNet B0	0.7665	0.7191	0.8544	0.683	0.7793	0.7809	581	489	227	99
EfficientNet Bl	0.7636	0.7139	0.8588	0.6732	0.792	0.7797	584	482	234	96
MobileNet	0.7794	0.7657	0.7882	0.7709	0.7365	0.7768	536	552	164	144
ResNet34	0.8095	0.782	0.8441	0.7765	0.7957	0.8119	574	556	160	106
ResNet50	0.7958	0.7548	0.8603	0.7346	0.8283	0.8041	526	190	95	385
VGG	0.8109	0.7955	0.8235	0.7989	0.8184	0.8092	560	572	144	120

**Table 3:** Comparison between different readers.

Reader	Number of Images	The Correct Number of Images	Precision
Attending doctor1	200	181	90.50%
Attending doctor1 + CNN	200	192	96%
Attending doctor2	200	185	91%
Attending doctor2 + CNN	200	195	97.50%
Attending doctor3	200	183	90.20%
Attending doctor3 + CNN	200	194	97.00%
Chief1	200	195	97.50%
Chief2	200	191	95.50%

## Data Availability

The data presented in this study are available on request from the corresponding author.

## References

[1] Mordecai S.C. (2014). Treatment of meniscal tears: An evidence based approach. World J. Orthop..

[2] Ouyang X., Wei B., Hong S.D., Xin F., Wang L., Yang X.W., Wang L.M. (2015). Arthroscopic Characteristics of Normal and Discoid Meniscus Injury, and Efficiency of Recovery in Each Type of Meniscus Injury. Cell Biochem. Biophys..

[3] Blake M.H., Lattermann C., Johnson D.L. (2017). MRI and Arthroscopic Evaluation of Meniscal Injuries. Sport. Med. Arthrosc. Rev..

[4] Englund M., Roemer F.W., Hayashi D., Crema M.D., Guermazi A. (2012). Meniscus pathology, osteoarthritis and the treatment controversy. Nat. Rev. Rheumatol..

[5] Greif D.N., Baraga M.G., Rizzo M.G., Mohile N.V., Silva F.D., Fox T., Jose J. (2020). MRI appearance of the different meniscal ramp lesion types, with clinical and arthroscopic correlation. Skelet. Radiol..

[6] Stensby J.D., Pringle L.C., Crim J. (2021). MRI of the Meniscus. Clin. Sport. Med..

[7] Hoover K.B., Vossen J.A., Hayes C.W., Riddle D.L. (2020). Reliability of meniscus tear description: A study using MRI from the Osteoarthritis Initiative. Rheumatol. Int..

[8] Foreman S.C., Liu Y., Nevitt M.C., Neumann J., Joseph G.B., Lane N.E., McCulloch C.E., Link T.M. (2021). Meniscal Root Tears and Extrusion Are Significantly Associated with the Development of Accelerated Knee Osteoarthritis: Data from the Osteoarthritis Initiative. Cartilage.

[9] Jeon S.W., Jung M., Choi C.H., Kim S.G., Kim S.H. (2021). Factors Related to Meniscal Extrusion and Cartilage Lesions in Medial Meniscus Root Tears. J. Knee Surg..

[10] Badlani J.T., Borrero C., Golla S., Harner C.D., Irrgang J.J. (2013). The effects of meniscus injury on the development of knee osteoarthritis: Data from the osteoarthritis initiative. Am. J. Sports Med..

[11] Hare K.B., Stefan L.L., Kise N.J., Risberg M.A., Roos E.M. (2017). Middle-aged patients with an MRI-verified medial meniscal tear report symptoms commonly associated with knee osteoarthritis. Acta Orthop..

[12] Lassau N., Estienne T., de Vomecourt P., Azoulay M., Cagnol J., Garcia G., Majer M., Jehanno E., Renard-Penna R., Balleyguier C. (2019). Five simultaneous artificial intelligence data challenges on ultrasound, CT, and MRI. Diagn. Interv. Imaging.

[13] Ryzewicz M., Peterson B., Siparsky P.N., Bartz R.L. (2007). The Diagnosis of Meniscus Tears. Clin. Orthop. Relat. Res..

[14] Lecouvet F., Van Haver T., Acid S., Perlepe V., Kirchgesner T., Vande Berg B., Triqueneaux P., Denis M.L., Thienpont E., Malghem J. (2018). Magnetic resonance imaging (MRI) of the knee: Identification of difficult-to-diagnose meniscal lesions. Diagn. Interv. Imaging.

[15] Kocabey Y., Tetik O., Isbell W.M., Atay Ö.A., Johnson D.L. (2004). The value of clinical examination versus magnetic resonance imaging in the diagnosis of meniscal tears and anterior cruciate ligament rupture. Arthroscopy J. Arthrosc. Relat. Surg..

[16] Phelan N., Rowland P., Galvin R., O’Byrne J.M. (2016). A systematic review and meta-analysis of the diagnostic accuracy of MRI for suspected ACL and meniscal tears of the knee. Knee Surg. Sport. Traumatol. Arthrosc..

[17] Kunze K.N., Rossi D.M., White G.M., Karhade A.V., Deng J., Williams B.T., Chahla J. (2021). Diagnostic Performance of Artificial Intelligence for Detection of Anterior Cruciate Ligament and Meniscus Tears: A Systematic Review. J. Arthrosc. Relat. Surg..

[18] Haggenmüller S., Maron R.C., Hekler A., Utikal J.S., Barata C., Barnhill R.L., Beltraminelli H., Berking C., Betz-Stablein B., Blum A. (2021). Skin cancer classification via convolutional neural networks: Systematic review of studies involving human experts. Eur. J. Cancer.

[19] Esteva A., Kuprel B., Novoa R.A., Ko J., Swetter S.M., Blau H.M., Thrun S. (2017). Dermatologist-level classification of skin cancer with deep neural networks. Nature.

[20] Sugeno A., Ishikawa Y., Ohshima T., Muramatsu R. (2021). Simple methods for the lesion detection and severity grading of diabetic retinopathy by image processing and transfer learning. Comput. Biol. Med..

[21] Chamberlin J., Kocher M.R., Waltz J., Snoddy M., Stringer N.F.C., Stephenson J., Sahbaee P., Sharma P., Rapaka S., Schoepf U.J. (2021). Automated detection of lung nodules and coronary artery calcium using artificial intelligence on low-dose CT scans for lung cancer screening: Accuracy and prognostic value. BMC Med..

[22] Tan T., Wang Z., Du H., Xu J., Qiu B. (2021). Lightweight pyramid network with spatial attention mechanism for accurate retinal vessel segmentation. Int. J. Comput. Assist. Radiol. Surg..

[23] Li Z., Lang C., Liew J.H., Li Y., Hou Q., Feng J. (2021). Cross-Layer Feature Pyramid Network for Salient Object Detection. IEEE Trans. Image Process..

[24] Crawford R., Walley G., Bridgman S., Maffulli N. (2007). Magnetic resonance imaging versus arthroscopy in the diagnosis of knee pathology, concentrating on meniscal lesions and ACL tears: A systematic review. Br. Med. Bull..

[25] Roblot V., Giret Y., Bou Antoun M., Morillot C., Chassin X., Cotten A., Zerbib J., Fournier L. (2019). Artificial intelligence to diagnose meniscus tears on MRI. Diagn. Interv. Imaging.

[26] Shin H., Choi G.S., Shon O., Kim G.B., Chang M.C. (2022). Development of convolutional neural network model for diagnosing meniscus tear using magnetic resonance image. BMC Musculoskelet. Disord..

[27] Fritz B., Marbach G., Civardi F., Fucentese S.F., Pfirrmann C.W.A. (2020). Deep convolutional neural network-based detection of meniscus tears: Comparison with radiologists and surgery as standard of reference. Skelet. Radiol..

